# Quantitative assessment of parkinsonian bradykinesia based on an inertial measurement unit

**DOI:** 10.1186/s12938-015-0067-8

**Published:** 2015-07-12

**Authors:** Houde Dai, Haijun Lin, Tim C Lueth

**Affiliations:** Quanzhou Institute of Equipment Manufacturing, Fujian Institute of Research on the Structure of Matter, Chinese Academy of Sciences, Quanzhou, China; Institute of Micro Technology and Medical Device Technology, Faculty of Mechanical Engineering, Technische Universitaet Muenchen, Boltzmannstr. 15, 85748 Garching, Germany; College of Polytechnic, Hunan Normal University, Changsha, China

## Abstract

**Background:**

As the most characteristic feature of Parkinson’s disease (PD), bradykinesia (slowness of movement) affects all patients with Parkinson’s disease and interferes with their daily activities. This study introduces a wearable bradykinesia assessment system whose core component is composed of an inertial measurement unit.

**Methods:**

The system diagram and assessment task were defined in accordance with clinical requirements from neurologists. Based on hand grasping actions, calculations of hand grasping ranges and statistical methods of quantitatively assessing parkinsonian bradykinesia were presented. Seven control subjects and eight patients were tested with this system.

**Results:**

Experimental results show that a calculated bradykinesia parameter (modified mean range, instead of mean and standard deviation of the grasp ranges) correlated well with the evaluations of a neurologist (Pearson’s correlation coefficient *r* = −0.83, *p* < 0.001).

**Conclusions:**

The bradykinesia assessment system was tested on both health subjects and PD patients. The results show that this system has greater correlation with the evaluations by neurologists than other parkinsonian bradykinesia assessment systems. The modified mean range was verified as the major bradykinesia parameter (key indicator). This study is helpful to those who want to use consumer-grade inertial sensors for quantitative assessment of motor symptoms during treatment.

## Background

Parkinson’s disease (PD) is a movement disorder, which denotes degenerative and progressive disorder of the central nervous system [1]. Parkinson’s disease affects patients in many different ways with a variety of symptoms, such as tremor, bradykinesia, rigidity, and postural instability [2].

Parkinsonian tremor is the most well-known and apparent symptom. However, around 30% of PD patients are not affected by tremor, while Parkinsonian bradykinesia appears in almost all PD patients [2]. Parkinsonian bradykinesia (slowness of motion) involves difficulties in planning, beginning, and executing movement; and difficulties in performing sequential and simultaneous tasks [3].

In the clinical setting, a series of joint tasks, such as finger tapping, whole hand grasping, and supination-pronation movements of hands, are performed by the patients to assess the bradykinesia severity. A neurologist assesses the severity of parkinsonian bradykinesia according to clinical ratings [4, 5].

The current standard for evaluating parkinsonian bradykinesia is the Unified Parkinson’s Disease Rating Scale (UPDRS) [4], a qualitative assessment that is completed by the subjective judgment of neurologists. The symptoms are rated on a scale from 0 to 4 (0: normal; 1: slight; 2: mild; 3: moderate; and 4: severe). However, this experience-based assessment may differ among different examiners [2].

To improve the reliability of assessment, some research groups have focused on quantitative assessment of bradykinesia based on computer systems and motion sensors, such as magnetic sensors, electromagnetic sensors, touch sensors, gyroscopes, and accelerometers [5–18]. An overview of recent approaches in parkinsonian bradykinesia assessment is given in Table 1. Based on the examiner’s eyesight or sensors, different bradykinesia parameters were adopted. For example, Niazmand et al. used two metal contact pieces, which worked as a touch sensor, to measure the interval of finger taps. However, this self-made touch sensor had long wires and had big live contact pieces [7].

**Table 1:** Summary of bradykinesia assessment methods

Method or research	Joint; task	Angle measure	Parameters
*Examiners’ assessment*	*Hand; grasps, P*–*S* ^*a*^ */fingers; taps*	*Visual by the neurologist*	*Range, frequency*
Salarian [6]	Wrist; –	Gyroscope	Range, amplitude, durations
Niazmand [7]	Fingers; taps	Metal contact pieces	Average and standard deviation of the durations
Kim [8]	Fingers; taps	Gyroscope	RMS velocity and angle, PSD
Espay [5]	Fingers; taps	Gyroscope	Speed, amplitude, rhythm
Heldman [9]	Foot; toe-taps, leg agility	Gyroscope	RMS of angular velocity and angle
Heldman [10], Kinesia HomeView™ [14]	*Hand; grasps, P*–*S* ^*a*^ */fingers; taps*	Gyroscope, accelerometer	Range, frequency
Printy [11]	Finger; taps/hand; P–S^a^	Gyroscope, accelerometer	Frequency, amplitude, rhythm
Dunnewold [12]	Wrist; Taps	Accelerometer	Tap rate, durations
Zwartjes [13]	Wrist/thigh/foot/sternum; taps	Accelerometer	Range and minimum of acceleration, durations,

*P–S* ^*a*^ Pronation–Supination, *RMS* root mean square and *PSD* power spectral density.

Most recent studies are based on MEMS (micro-electro-mechanical system) inertial sensors [5–18]. Accelerometers, gyroscopes, magnetometers, and their combinations, i.e. inertial measurement units, are the fundamental inertial sensors [19].

Salarian et al. presented a gyroscope-based ambulatory system for quantitative assessment of wrist bradykinesia. There was a significant Pearson’s correlation (*r* = −0.73 to −0.83, *p* = 0.001) between the estimated bradykinesia parameters (range, amplitude, and periods of movements) and UPDRS bradykinesia scores [6]. Kim et al. quantified bradykinesia during finger taps by using a gyroscope. RMS (root-mean-square) velocity, RMS angle, and the estimated power around the dominant frequency were correlated well with clinical finger tapping scores (*r* = −0.73 to −0.80, *p* = 0.001) [8]. Heldman et al. presented a leg assessment system of PD by using inertial sensors, with an average correlation coefficient of 0.86 [9]. Heldman et al. also developed a hand bradykinesia assessment system, whose correlation coefficient is 0.67 [10]. Printy et al. presented a smart phone application for quantifying finger bradykinesia (*r* = −0.48, *p* = 0.04) [11]. Dunnewold et al. tested subjects with wrist taps (*r* = −0.61 to −0.87) [12]. Zwartjes et al. designed an accelerometer-based ambulatory monitoring system to classify bradykinesia severity simultaneously (*r* = −0.57 to −0.71) [13].

Currently, there has been no unique parameter for parkinsonian bradykinesia. The parkinsonian bradykinesia assessment systems provide multiple parameters such as range, frequency, and velocity as bradykinesia parameters [5–18]. Thus, it is difficult for doctors or patients to judge the bradykinesia severity. For the patients and doctors, a single parameter with the highest correlation with the UPDRS bradykinesia scores is preferable. In addition, some of these systems have large dimensions or complex operations. Furthermore, some of their assessment tasks are difficult for old people to perform.

Bradykinesia encompasses slowness, decreased movement amplitude, and dysrhythmia. It means the inability to generate maximum speed, power, or force. These movement tasks are suitable to be tracked by MEMS inertial sensors [20]. Therefore, the goal of this study was to develop a portable system for use both inside and outside the operating room based on the newly developed MEMS inertial sensors [18, 19]. In addition, we also defined a dominant bradykinesia parameter (key indicator) to represent the severity of parkinsonian bradykinesia. A preliminary version of this paper has been reported [18].

## Methods

In this section, the system diagram of the bradykinesia assessment system is firstly introduced, and the preceding methods and prototype implementation are presented.

### System diagram and assessment task

**Figure 1:**
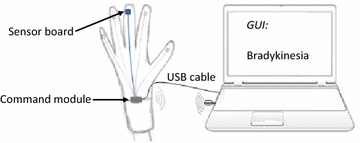
System diagram of the bradykinesia assessment system. The command module, which includes a microcontroller and serial-USB interface, acquires inertial sensor data and sends them to the computer. Doctors can operate the system and view the assessed bradykinesia scores, which are displayed on the graphical user interface (GUI). Additional wireless communication can be adopted for further applications.

Figure 1 shows the system diagram of the bradykinesia assessment system. The wired communication was adopted due to the requirements of the operating room. The sensor signals from the command module are sent to the computer for further processing [21]. The finger’s activity is tracked by an inertial measurement unit (IMU) on the top side of the patient’s middle finger, which includes a three-axis gyroscope and a three-axis accelerometer.

A 10-second whole-hand grasp task was chosen as the assessment action after discussion with neurologists [18]. A single closing and opening action of the fingers is regarded as a grasp cycle. The subject is required to grasp with the greatest possible frequency and range for 10 s.

### Processing method

The hand grasping angles obtained from patients with mild bradykinesia have a consistent amplitude and frequency, and appear sinusoidal. However, hand grasping angles of patients with severe bradykinesia have much lower and more inconsistent amplitudes and frequencies. Speeds, amplitudes, halts, hesitations, and any decline in amplitude are evaluated.

In order to acquire orientation values in real time, a complementary filter, i.e. the direction cosine matrix method (DCM), was adopted [22].

**Figure 2:**
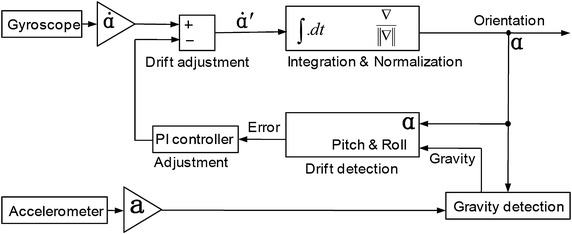
Block diagram of DCM algorithm. Here PI controller denotes a proportional-integral controller. The proportional gain and integral gain for both patch and roll were 0.0125 and 9e-6, respectively. For the six-axis DCM algorithm, there is no yaw-axis input for the drift detection.

DCM is another way, other than Euler angles, to construct a rotation matrix. The traditional Euler angles have drawbacks such as a gimbal lock. The six-axis DCM algorithm is based on an IMU. The gyroscope is used as the primary source of orientation information. The accelerometer is used for roll–pitch drift correction because it has no drift over time. Only the gravity vector of the accelerometer is used for the drift detection. As shown in Figure 2, a proportional plus integral (PI) controller is used for adjusting the drifts [23]. Each of the rotational drift correction vectors (roll and pitch) is multiplied by weights and fed to a PI feedback controller.

As a control loop feedback mechanism (controller) widely used in industrial control systems, the PI controller calculates an error value as the difference between a measured process variable and a desired set-point. The controller attempts to minimize the error by adjusting the process through the use of a manipulated variable.

After that, the drift adjustments are added to the gyroscope vectors to produce corrected gyroscope vectors. The outputs of the algorithm are three-dimensional angles (orientation).

The grasping ranges (**α**_**PP**_ or ***φ***) are the three-dimensional peak-to-peak values, which are calculated separately, during grasping cycles of a bradykinesia task [10]. The combined triple-axis grasping range (|***φ***|) is the sum of the three-axis grasping ranges (***φ***).

The number of peak-to-peak angle values during the 10-s assessment period is related to the dominant frequency of hand grasps. The mean value of peak-to-peak angle values (grasp ranges) represents the amplitude of bradykinesia. The standard deviation (SD) value of grasp ranges represents the change of amplitude during the grasping task. According to the requirements of neurologists, the mean and SD of the grasp ranges represent the severity of a parkinsonian bradykinesia.

**Figure 3:**
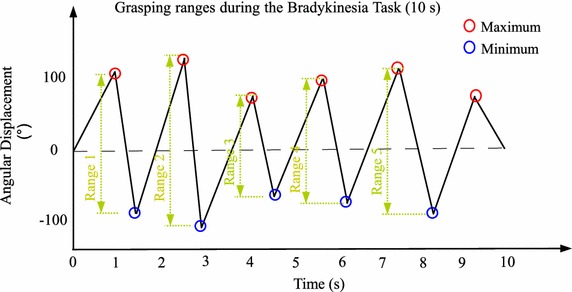
Peak detection for grasping ranges in a bradykinesia task. After the maximum and minimum points of the grasping ranges are found, the grasping ranges during the one-time bradykinesia task are acquired.

Figure 3 explains the peak-detection method during bradykinesia quantification. There are five hand-grasping cycles in this figure. A peak-detection algorithm could be used to calculate peak-to-peak angle values (ranges 1–5: |***φ***|_1_–|***φ***|_5_).

**Figure 4:**
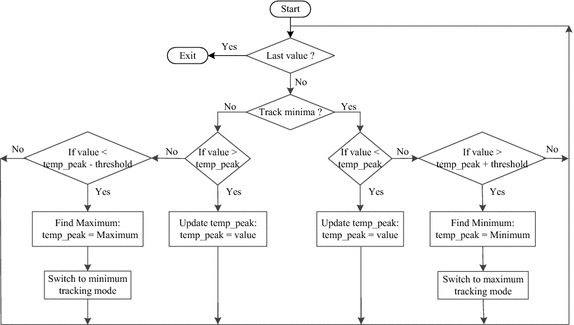
Flowchart of the peak-detection algorithm. The algorithm is performed for the switching between the minimum and maximum tracking modes. The behaviour of only saving a peak, when a threshold has been exceeded, ensures that the algorithm does not categorize small peaks of noise as signal peaks.

The flowchart of the peak-detection algorithm is shown in Figure 4. By proceeding through the data starting at zero, the peak detection algorithm tracks the minima and the maxima of the hand-grasping cycles [24].

Tremors occur during the bradykinesia assessment task and it is difficult to separate them in the frequency domain. An angular displacement threshold, for example ±20° for both maximum and minimum, can be used to remove the unnecessary peak points. For most subjects, the grasp ranges are greater than 20 degrees, so the threshold was fixed at 20°.

The mean and SD values of hand grasping ranges are easy to calculate by using statistical methods. Equations 1 and 2 show the mathematical formulas used to determine ($\overline{{\left| \varvec{\varphi } \right|}}$) and $\sigma_{\varphi }$:

1 $$\overline{{\left| \varvec{\varphi } \right|}} = \left( {\mathop \sum \limits_{i = 1}^{N} \left| \varvec{\varphi } \right|_{i} } \right)/N,$$

2 $$\sigma_{\left| \phi \right|} = \sqrt {\frac{1}{N - 1}{ \cdot }\mathop \sum \limits_{i = 1}^{N} \left( {\left| \varvec{\varphi } \right|_{i} - \overline{{\left| \varvec{\varphi } \right|}} } \right)^{2} } ,$$

where |***φ***|_*i*_ is the combined hand grasping range (peak-to-peak values) in a single grasp cycle; and *N* is the number of hand grasping cycles in a 10-s bradykinesia task.

The dominant frequency of hand grasps is calculated by using the Fast Fourier Transform (FFT) method from the grasping ranges.

In addition, the difficulty in selecting or activating motor programs in the central nervous system may result in akinesia (inability to initiate movement) in the patient’s daily life. Akinesia (absence of movement) during the bradykinesia task is the delay (action time) of the patient in starting the assessment task after receiving the instruction from the examiner [25]. In this study, the effect of akinesia on the parkinsonian bradykinesia was not considered.

### Prototypical realization

**Figure 5:**
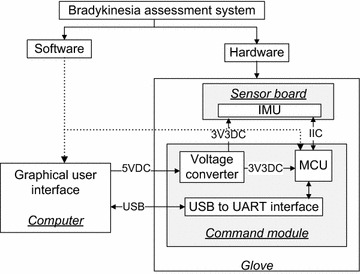
Internal architecture (hardware and software) of the bradykinesia assessment system. The power supply and data communication of the system are indicated. The software includes the programs, in both the computer and microcontroller (MCU).

The system implementation of the bradykinesia assessment system is shown in Figure 5.

The full ranges of the IMU in the parkinsonian bradykinesia assessments are listed as follows [14]:

- Angular velocity: ±2,000°/s (degrees per second or dps) in three dimensions;
- Acceleration: ±4*g* in each axis, here *g* is the gravitational acceleration (1*g* = 9.8 m/s^2^).

**Figure 6:**
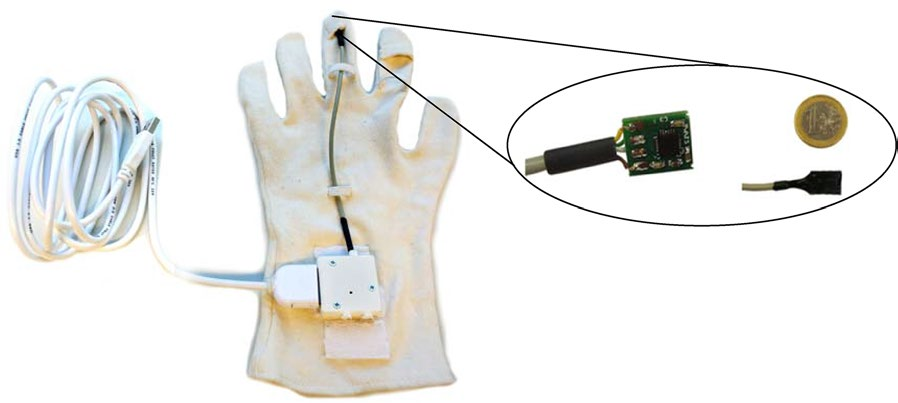
Photos of the prototype for the bradykinesia assessment system. The command module was 34 mm × 32 mm × 7 mm. Both the command module and sensor board had indirect contact with the human body. The sensor board (dimensions: 14 mm × 11 mm × 2 mm) is shielded by a synthetic rubber coating material.

MPU6050 (Invensense Inc., USA), an inertial measurement unit which is used for motion tracking, was chosen in this project. The axes of the accelerometer and gyroscope are the same [26]. Thus adjustment of the axes before sensor fusion is not necessary. The final implementation of the bradykinesia assessment system is shown in Figure 6. A shielded four-pin cable was used to connect the sensor board to the command module.

The USB port of the computer provided the power supply (+5 V) for the command module. The IMU in the sensor board was connected to the microprocessor via the IIC (pronounced I-two-C) serial interface. The microprocessor sampled the IMU data at 100 Hz.

The data transmission, signal processing, and GUI of the bradykinesia assessment system were carried out on a computer using LabVIEW 2011 (National Instruments Corp., USA).

**Figure 7:**
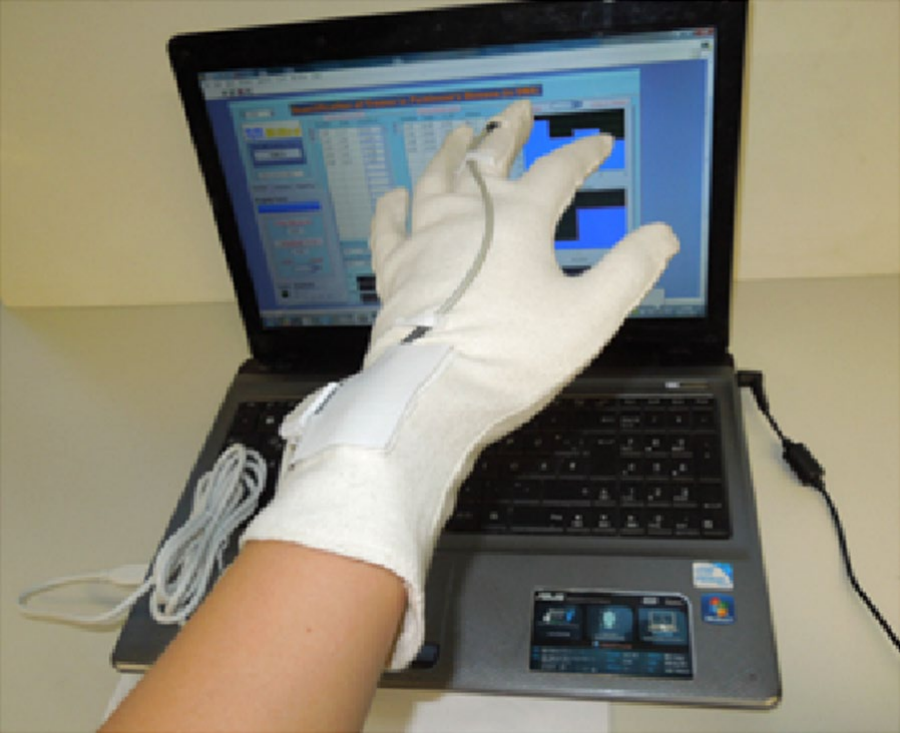
Bradykinesia assessment system. This system is based on a computer, a textile glove, and a command module.

The operation of the bradykinesia assessment system is shown in Figure 7.

## Experiment of bradykinesia assessment

In this section, the clinical experiment and the results are presented. The prototype was tested with both healthy controls and PD patients.

### Hypothesis

We expected the correlations of bradykinesia parameters (mean and SD values of amplitude, and grasp frequency) between the bradykinesia assessment system and clinical ratings to meet the following requirement [5–18]:

*H*_0_: Pearson’s correlation coefficient *r* = 0.79; *H*_1_: *r* ≠ 0, *α* = 0.01 (2-tailed).

### Materials

- A bradykinesia assessment system;
- A computer installed with the GUI of the bradykinesia assessment system (based on LabVIEW, National Instruments Corp., USA) and MATLAB (Mathworks Inc., USA).

### Experiment setup

Seven healthy controls (average age: 57. 1 ± 21. 1 years) and nine PD patients (average age: 72.8 ± 10.0 years) were required to execute hand grasping actions as widely and quickly as possible for 10 s, and repeat the assessment task two or three times for each subject. The first time of each assessment task was discarded for training. However, a severe PD patient (UPDRS bradykinesia score *D* = 4) was unable to perform the bradykinesia task. The patients were tested 24 h after off medication and without deep-brain stimulation. The one-time whole-hand grasping task included several grasp cycles, which was the number of peak-to-peak ranges.

An IMU, attached to the middle finger, was used to measure the angular displacement of the middle finger during the bradykinesia assessment task. The bradykinesia parameters, calculated by the assessment system and used as the severity features of bradykinesia, were compared to the ratings of a neurologist [18].

The mean and SD values of the grasp ranges represent the severity of the parkinsonian bradykinesia. However, we inferred that the dominant frequency also has a strong correlation with the UPDRS score; thus a modified mean range, which equals the product of the dominant frequency and mean range during a single bradykinesia assessment task, was chosen as the dominant bradykinesia parameter.

The UPDRS scores for healthy controls are judged as zero, although their assessed values may deviate from normal values.

Our research group previously adopted finger tapping as assessment task. However, neurologists suggested that we adopt whole-hand grasping. During clinical experiments, therefore, the patient and control subjects were required to perform both finger tapping and whole-hand grasping actions. Most patients and control subjects preferred whole-hand grasping as it was easier for them to perform.

## Discussion

**Figure 8:**
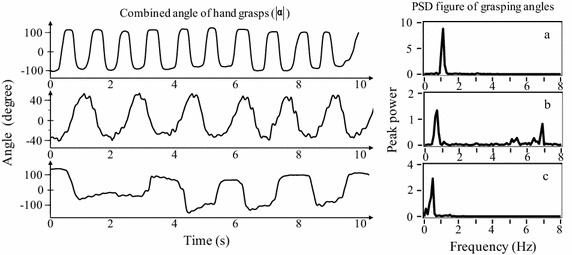
Ten-second hand grasps and their PSD figures. **a** 72 year old control subject; **b** 82 year old PD patient with tremor and bradykinesia, UPDRS bradykinesia score *D* = 1; **c** 86 year old PD patient, UPDRS bradykinesia score *D* = 3. The peak powers in the figure were calculated based on the power estimation around the dominant frequency and with a weighted scale.

Figure 8 shows three 10-s waveforms of hand grasps and their power spectral density (PSD) figures. Figure 8a shows the waveforms from a healthy subject, while Figure 8b, c show the waveforms from PD patients.

In addition, other motor symptoms also appeared in the hand grasp tasks. As shown in Figure 8b, action tremor appeared among some patients during the bradykinesia assessment task. As shown in Figure 8c, akinesia (difficulty initiating movement) appeared in a patient with severe bradykinesia [27]. It can be seen from Figure 8c that there was a delay time before he performed hand grasps continuously. As Figure 8c shows, the akinesia affects the cycles of hand grasps, thus the dominant frequency was reduced.

**Figure 9:**
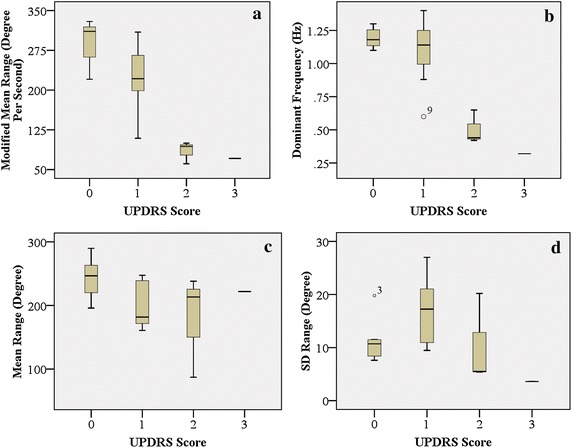
Box-plots displaying the relations between the judgments of a neurologist and the UPDRS bradykinesia parameters. **a** Modified mean range; **b** Dominant frequency; **c** Mean range of the grasping ranges; **d** SD of the grasping ranges. The unit of ranges in all plots is degrees.

Table 2 shows the calculated bradykinesia parameters of this experiment and the Pearson’s correlation coefficients (*r*) between these parameters and the judgment of the neurologist (UPDRS scores *D*). Figure 9 shows the relations between the judgments of a neurologist and the UPDRS bradykinesia parameters.

**Table 2:** Clinical experiment results

Subjects; age (years)	Dominant frequency	Mean range	SD ranges	Modified mean range	UPDRS score (*D*)
Control 1; 72	1.11 Hz	202.9°	7.6°	220.5°/s	0
Control 2; 46	1.29 Hz	255.4°	11.5°	329.5°/s	0
Control 3; 29	1.10 Hz	289.8°	19.8°	318.8°/s	0
Control 4; 34	1.30 Hz	237.2°	10.7°	310.7°/s	0
Control 5; 61	1.18 Hz	270.9°	11.5°	319.7°/s	0
Control 6; 76	1.16 Hz	246.6°	8.6°	286.0°/s	0
Control 7; 82	1.22 Hz	195.8°	8.2°	238.5°/s	0
Patient 1; 82	0.88 Hz	241.6°	10.2°	212.6°/s	1
Patient 2; 73	1.34 Hz	162.6°	20.0°	226.0°/s	1
Patient 3; 76	1.40 Hz	182.0°	11.7°	263.9°/s	1
Patient 4; 75	1.13 Hz	160.8°	16.8°	184.9°/s	1
Patient 5; 56	1.16 Hz	247.5°	17.7°	309.4°/s	1
Patient 5; 56	1.11 Hz	236.4°	22.1°	267.1°/s	1
Patient 6; 61	1.15 Hz	180.7°	27.0°	216.8°/s	1
Patient 6; 61	0.65 Hz	87.1°	5.5°	61.0°/s	2
Patient 7; 73	0.60 Hz	181.7°	9.5°	109.0°/s	2
Patient 7; 73	0.44 Hz	213.3°	20.2°	93.6°/s	2
Patient 8; 86	0.42 Hz	238.0	5.4°	100.0°/s	2
Patient 8; 86	0.32 Hz	221.8°	3.6°	71.0°/s	3
*r*	−0.79	−0.39	−0.13	−0.83	
*p* (2-tailed)	<0.001	0.10	0.59	<0.001

The seven control subjects had a dominant frequency at 1.19 ± 0.08 Hz and mean range at 24,246.7° ± 34.2°. For patients with slight bradykinesia, the dominant frequency was at 1.17 ± 0.17 Hz and mean range at 195.0° ± 37.6°, while the patients with mild and severe bradykinesia were 0.54° ± 0.16 Hz and 188.4° ± 60.2° respectively.

As Table 2 shows, the patients with severe symptoms (Patients 6–8) executed grasping cycles with a lower modified mean range (187.1° ± 53.9°) and dominant frequency (0.60 ± 0.30 Hz), while the modified mean range and dominant frequency from control subjects were 246.3° ± 36.2° and 1.2° ± 0.1° respectively. The dominant frequency of hand grasps and the modified mean range had the highest correlations with the UPDRS bradykinesia score (*r* = −0.79 and −0.83 respectively; *p* < 0.001). The correlations were greater than 0.81, which was defined by the Kinesia system, thus *H*_*1*_ was accepted [10].

However, the mean range of hand grasps had a low correlation with the UPDRS bradykinesia score (*r* = −0.39). For the SD ranges, there was an even smaller correlation coefficient (*r* = −0.13) with the UPDRS ratings in this experiment. The mean and SD of the grasping ranges should therefore not be regarded as major bradykinesia parameters.

As Table 2 also shows, the age of a subject may influence her or his performance of hand grasping, as shown by Control subject 1 and Patient 5, whose mean range deviated from the normal situation. Control subject 1 (72 years) could not perform great amplitudes (mean range = 202.9°) due to her age, while Patient 5 (56 years, UPDRS score = 1) performed with a higher dominant frequency (1.14 Hz) and mean range (242.0°).

As a conclusion, the dominant frequency and modified mean range between the bradykinesia assessment system and a surgeon were significant (|*r*| ≥ 0.83). Thus the modified mean range can be defined as the major parkinsonian bradykinesia parameter.

## Conclusions

A MEMS IMU, which is a typical motion sensor, was employed in a wearable bradykineisa assessment system and performed motion tracking.

Instead of finger tapping for bradykinesia assessment as in most research groups, this study uses whole-hand grasping. Compared with the more common used finger tapping, hand grasping is easy for patients to perform.

There were great differences between the control subjects and patients with parkinsonian bradykinesia, because the average modified mean range of control subjects, patients with slight, and mild or worse bradykinesia were 294.9, 240.1, and 86.92°/s respectively.

At the beginning of this study, neurologists considered the mean and SD values of grasping ranges to have an even higher correlation with the bradykinesia scores. According to the results of the clinical experiment, however, the dominant frequency and modified mean range of hand grasps correlated well with the UPDRS ratings (the absolute values of correlation coefficients were 0.79 and 0.83 respectively), while the mean and SD of grasping ranges were 0.39 and 0.13 respectively. As a result, the modified mean range had the highest correlation coefficient with the judgment of the neurologist and can thus be adopted as the major bradykinesia parameter.

Future research will include carrying out the experiment with more PD patients. However, attention must be paid to the data processing methods as concerns the following:

1. Verification and comparison of the different calculation methods of the hand grasping range because calibration procedures, signal filters, and multi-sensor algorithms play a key role in the range calculation [27].
2. As there are no previous studies about the correlation between age and the quantitative parameters of parkinsonian bradykinesia, the repeatability of bradykinesia parameters and effects of the subject’s age and physical status (both mental and physical factors) on the bradykinesia parameters should be investigated [28].
3. Effects of PD’s other symptoms, such as tremor, akinesia, and motor fluctuations [29, 30], on the bradykinesia parameters.
4. Correlations between the features were taken into consideration together with the clinical evaluations of bradykinesia provided by several neurologists, in order to assess the robustness of the method proposed.
5. The angular displacement threshold was fixed at 20 degrees. In future works, we can set an automatic threshold which is proportional to the grasp ranges.
